# Prediction of Epidemic Spread of the 2019 Novel Coronavirus Driven by Spring Festival Transportation in China: A Population-Based Study

**DOI:** 10.3390/ijerph17051679

**Published:** 2020-03-04

**Authors:** Changyu Fan, Linping Liu, Wei Guo, Anuo Yang, Chenchen Ye, Maitixirepu Jilili, Meina Ren, Peng Xu, Hexing Long, Yufan Wang

**Affiliations:** 1 School of Sociology, Central China Normal University, Wuhan 430079, Hubei Province, China; fanchangyu@mail.ccnu.edu.cn (C.F.); wangyufanberkeley@berkeley.edu (Y.W.); 2 School of Public Administration, Hangzhou Normal University, Hangzhou 311121, Zhejiang Province, China; 3 School of Social and Behavioral Sciences, Nanjing University, Nanjing 210046, Jiangsu Province, China; weiguo@nju.edu.cn (W.G.); dg1907020@smail.nju.edu.cn (A.Y.); yechenchen93@gmail.com (C.Y.); dg1907012@smail.nju.edu.cn (M.J.); rmn_sysu@foxmail.com (M.R.); 4 School of Sociology, Wuhan University, Wuhan 430072, Hubei Province, China; allahx1895@hotmail.com; 5 School of Economics, Minzu University of China, Beijing 100081, China; longhexing@muc.edu.cn

**Keywords:** 2019 novel coronavirus, the origins of Wuhan’s migrants, population size, social characteristics of floating population, quasi-experimental approach

## Abstract

After the 2019 novel coronavirus (2019-nCoV) outbreak, we estimated the distribution and scale of more than 5 million migrants residing in Wuhan after they returned to their hometown communities in Hubei Province or other provinces at the end of 2019 by using the data from the 2013–2018 China Migrants Dynamic Survey (CMDS). We found that the distribution of Wuhan’s migrants is centred in Hubei Province (approximately 75%) at a provincial level, gradually decreasing in the surrounding provinces in layers, with obvious spatial characteristics of circle layers and echelons. The scale of Wuhan’s migrants, whose origins in Hubei Province give rise to a gradient reduction from east to west within the province, and account for 66% of Wuhan’s total migrants, are from the surrounding prefectural-level cities of Wuhan. The distribution comprises 94 districts and counties in Hubei Province, and the cumulative percentage of the top 30 districts and counties exceeds 80%. Wuhan’s migrants have a large proportion of middle-aged and high-risk individuals. Their social characteristics include nuclear family migration (84%), migration with families of 3–4 members (71%), a rural household registration (85%), and working or doing business (84%) as the main reason for migration. Using a quasi-experimental analysis framework, we found that the size of Wuhan’s migrants was highly correlated with the daily number of confirmed cases. Furthermore, we compared the epidemic situation in different regions and found that the number of confirmed cases in some provinces and cities in Hubei Province may be underestimated, while the epidemic situation in some regions has increased rapidly. The results are conducive to monitoring the epidemic prevention and control in various regions.

## 1. Introduction

The outbreak of a new coronavirus (2019-nCoV) has spread internationally since the initial report of cases by Wuhan Municipal Health Commission, China on 31 December 2019 [1,2,3,4]. On 26 January 2020, WHO announced that there is a high risk of a 2019-nCoV epidemic in China and at a global level [5].

By 31 January 2020, 9720 confirmed cases, 15,238 suspected cases and 176 cures of 2019-nCoV, and 213 deaths from 2019-nCoV had been reported by the national authorities in China [6]. Some confirmed cases had also been reported in Thailand, Singapore, the United States, Australia, Japan, Korea, Malaysia, France, Vietnam, Nepal, Canada, Cambodia, Germany, and Sri Lanka [7].

Based on preliminary judgments, the transmission of this coronavirus has particular distinguishing features [8,9,10,11]. First, the epidemic first originated in Wuhan and then spread to other places [12,13,14,15,16]. Second, the illness can spread from human to human [9,13,14,15,16,17,18,19,20,21,22], for example, there have been several cases of human-to-human transmission in Guangdong Province during family gatherings [23], and several healthcare workers were diagnosed in the early period of the outbreak [24]. Additionally, the 2019-nCoV coronavirus can survive in an environment and on the surface of objects up to 14 days [8]. 

The floating population, i.e., the seasonal and temporary non-resident population of a geographic area, is a potential carrier for virus transmission [16,21]. Wuhan, the epicentre of the outbreak, has a large floating population, and, being in the centre of China, is a major travel hub. Wuhan is a megacity, with a population of over 11 million in 2018, which includes a floating population of 2,243,700 living there for six months or more [25]. 

This floating population may have exposure history in Wuhan, and could contribute to the increase in transmission of 2019-nCoV. Considering the features of 2019-nCoV transmission and the large-scale regional travel that occurs over the Spring Festival holiday [26], the Wuhan government announced the implementation of travel restrictions on 23 January 2020 [27]. However, many migrant workers had already left Wuhan by this date, and returned to their hometowns in other areas of China [28]. This floating population may therefore have transmitted 2019-nCoV in their hometowns, especially those members of this population within Hubei Province.

The dualistic structure of urban and rural populations in China is likely to exacerbate the 2019-nCoV epidemic [29]. Many of Wuhan’s floating population who are from rural areas but work in the Wuhan metropolitan area may be susceptible. For example, Wuhan’s Huanan Seafood Wholesale Market, which is regarded as a possible point source of the virus, is less than 600 metres from the Hankou Railway Station [30]. This district and railway station is a hub of migrant worker life and transport. 

As individual members of the floating population must frequently work overtime hours, are vulnerable to having poor nutrition, and due to lack of resources have higher rates of poor health [29,31], they may have lower resistance to 2019-nCoV infection. Due to lower educational investment in this population, it may be more difficult for individuals to identify preventive healthcare information, which may cause misidentification of 2019-nCoV infection as the common cold or flu. Compounding this issue, most public medical resources are concentrated in cities but are relatively scarce in rural areas [29,31]. Therefore, prevention and treatment of 2019-nCoV in rural areas will be more challenging if new phases of the epidemic emerge.

To determine the influence of the floating population on the spread of 2019-nCoV, we used data from China Migrants Dynamic Survey (CMDS) released by the National Health Commission of China to analyse the national distribution of the origins of Wuhan’s floating population, and provide reference information to predict the trend of the epidemic. 

The purposes of this study were to estimate the distribution and scale of the migrants residing in Wuhan after they returned to their hometown communities in Hubei Province or other provinces after the 2019-nCoV outbreak. The social characteristics of the migrants are also analysed in the study, including age group, educational level, pattern of migration, number of migrating family members per household, type of household registration, and reasons for migration. In addition, we assumed a quasi-experimental approach to estimate the epidemic prevention and control in various regions of China. On the one hand, these findings are conducive to epidemic prevention and control, and on the other hand, currently, the predictive model of the 2019-nCoV is not detailed enough because of the lack of relevant parameters. Therefore, this study is beneficial for virologists, epidemiologists and medical professionals to deepen the study of the virus transmission and improve the accuracy of model prediction. 

The analysis object of this study was the floating population who have lived in Wuhan for more than one month. Short-term migrants and students were not included. Distinct from other models of the dynamics of this epidemic, we used the information of the respondents from the CMDS and their family members to further explore the origins of Wuhan’s migrant population, such as their returning destination, population characteristics, family structures and other metrics. This approach can provide practical solutions to prepare prevention strategies, and approaches to assess resources for treatment and containment of the epidemic.

## 2. Research in Context

### 2.1. Evidence before This Study 

The National Health Commission of China released a report on 27 January 2020 that stated that 2019-nCoV could be transmitted not only via respiratory droplets, but also via direct contact. 2019-nCoV has now spread nationally and worldwide, and due to the lack of data on the size and origins of the floating population of Wuhan, it has been difficult for the Chinese government to arrange real-time medical resources and implement effective public health interventions.

### 2.2. Added Value of This Study 

We used data from the Wuhan Floating Population Monitoring Survey to estimate the size and origins of the migrant population in Wuhan. We also described the socio-demographic characteristics of this population, and compared confirmed cases from different regions to estimate the epidemic with modelling techniques. We found that three-quarters of Wuhan’s floating population are from Hubei Province, and that nearly 85% migrated with nuclear families. The number of members per family is 3 to 4, and most individuals are migrant workers from rural areas with low education levels. By comparing the predicted and actual values obtained from the model, we analysed the profile of the epidemic in various regions since January 25, and found that the spread of the 2019-nCoV has varied greatly between regions, and that the epidemic in some regions may be underestimated. There may also be unknowns, such as structural factors in some regions, that deserve further attention.

### 2.3. Implications of All the Available Evidence 

The majority of the floating population left Wuhan before the city was “closed off” by authorities, so our analysis will be useful for estimating the key geographic areas for prevention and control. The results indicate that the floating population of Wuhan is centred in Hubei Province and the surrounding provinces, so local government must quickly and effectively take steps to prevent further spread of 2019-nCoV. Higher-level governments must also strengthen the assistance they are providing, such as sending medical workers and medical supplies to these areas to avoid 2019-nCoV becoming a new pandemic. At the same time, it is important to increase surveillance in areas where the epidemic may be underestimated, and promptly identify prevention and control loopholes to reduce the burden of a new round of transmission. 

China has been deeply involved in the globalisation process, and even China’s central and western regions have become important links in the global production and trade chain. Therefore, while our research is aimed at China in the current era of migration, this research has practical implications for global public health and disease control, as floating populations are increasing in size all over the world and relationships between countries are becoming increasingly close. Thus, other countries should pay attention to the epidemic situation in specific geographic areas of China to prevent secondary and international transmission of the 2019-nCoV.

## 3. Methods

### 3.1. Data Sources

The data used in this study are based on the 2013–2018 China Migrants Dynamic Survey (CMDS), and the tabulated data of 5 million migrants in Wuhan recently released by the Wuhan municipal government. This survey was carried out via a multi-stage stratified sampling method, and collected data with structured questionnaires. As survey data is limited to the mainland provinces, municipalities, autonomous regions, and Hainan Province, the population analysed in this study excluded the populations of Hong Kong and Macao. A total of 11,999 samples of the resident floating population in Wuhan from 2013 to 2018 were extracted from the survey dataset.

According to the survey design, Wuhan’s floating population was defined as the population from other cities and districts, aged 15 and over, residing for more than one month in Wuhan, and not registered in Wuhan. In Table 1, the sample distribution of the resident population in Wuhan over time is presented. The sample size was 1999 in the year 2013, and 2000 for other years.

The outbreak is considered to have originated in the Huanan Seafood Market, near Hankou Railway Station in the Jianghan District, Wuhan, China. A severely infected area was concentrated in the urban areas of Hankou. From the sample distribution in Table 1, the floating population of Wuhan is seen to be concentrated in the urban area of Hankou. The results indicate that the sample proportion of Jianghan District and nearby Jiang’an District, Tongkou District, and Dongxihu District is approximately 51.6%. Therefore, the samples used in this study are suitably representative and thus acceptable for assessing the spread of the 2019-nCoV outbreak among the floating population.

We obtained data from the “Dingxiangyuan” National Real-time Epidemic website on confirmed cases from 25 January to 31 January 2020 [6]. These data are compiled from open data released by authorities such as the National Health Commission, and the provincial and municipal health commissions. To ensure data comparability, we collected the data daily from 12:00 to 14:00 every day.

### 3.2. Data Processing

Using the information of the floating population and their family members in Wuhan, we analysed their return destinations and their structural characteristics by descriptive statistical methods. In Table 2, the distribution of the origins of Wuhan’s floating population is presented, at the provincial level, over the past several years. The sample size is quite stable for each province over time. The province of origin for 75% of the floating population was Hubei Province, which contains the city of Wuhan, and approximately 25% of the population originated in other provinces.

The location information published in other historical survey data is limited to the province where household registration is located, due to a lack of data for 2019. The data provided by the Hubei Provincial Health Commission in 2017 includes more detailed information of prefectures, cities, districts and counties. The analysis was therefore divided into two parts; the first part comprised an analysis of the origin of Wuhan’s floating population at the provincial level using historical data. The second part comprised an analysis of the floating population within Hubei Province based on 2017 data. When analysing the floating population at the provincial level, we used all samples from the previous years—i.e., the mean of 6 years of data collection—to ensure the robustness of the results, in view of the stability of sample distributions in each province over time.

According to the current infectious features of 2019-nCoV, which are that middle-aged and elderly people have a high risk of infection, and transmission can occur between individuals, families and communities, we assessed several main variables. These comprised age group, educational level, pattern of migration, number of migrating family members per household, type of household registration, and reasons for migration. We defined these variables in the following ways: (1) age group was classified as under 20, 21–30, 31–40, 41–50, 51–60, and over 60; (2) educational level was divided into junior high school and below, high school/secondary school, and college and above; (3) pattern of migration was divided into independent migration, nuclear family migration, and extended family migration; (4) number of migrating family members per household was classified as 1, 2, 3, 4, and 5 or more; (5) types of household registration were divided into rural and urban household registration; (6) reasons for migration were working and doing business, family relocation, or other reasons.

### 3.3. Quasi-Experimental Design

The analyses assume a theoretical model of 2019-nCoV transmission. We considered a floating population of 5 million in Wuhan, who returned to their hometowns from 23 January 2020, as potential infected persons. Moreover, we added factors of demographic characteristics, the situation of medical diagnosis, government prevention and control, the number of confirmed cases, and undisclosed data to our statistical model to estimate the dynamics of the epidemic. After controlling for certain factors, we analysed the factors that were not controlled, such as government intervention and the number of statistical reports. 

Specifically, we first analysed the correlation between the size of the floating population in Wuhan and the number of confirmed cases per day. Then, we examined the differences among regions and proposed a transmission rate as a reference to compare the differences in regions. In the comparative analysis, we focused on the probably underestimated number of cases and the virus transmission rate to determine the likelihood of epidemics existing in different regions.

### 3.4. Prediction Model Setting

Finally, we predicted the floating population of Wuhan using statistical methods and compared it with the number of 2019-nCoV confirmed cases in each region, to identify regional differences of 2019-nCoV infection. Furthermore, we predicted the forthcoming epidemic trend at the prefecture- and province-level based on the proportion of Wuhan’s floating population represented by people from these areas. 

Human-to-human transmission of the 2019-nCoV has been confirmed. Four sets of factors that may influence regional differences appear to be involved: (1) Demographic factors, such as short-term business travellers between Wuhan and other regions, college students in Wuhan returning to their homes in other regions, Spring Festival tourists from Wuhan to other regions, and trans-regional floating populations for Spring Festival family reunions from or across Wuhan; (2) Intervention factors, such as medical treatments and governmental preventative measures; (3) Information disclosure and the information release system; and (4) Other unknown factors.

We considered all these factors, and hypothesised the social environment of 2019-nCoV transmission. First, although the government had taken the unprecedented measure of sealing off Wuhan city on 23 January 2020, we assumed that, at that time, the entire floating population of Wuhan, all short-term business travellers to Wuhan and all college students in Wuhan had returned to their hometowns throughout China, because 24 January 2020 was the Spring Festival’s Eve (normally, the 2020 Spring Festival holiday from January 24 to 30). Moreover, this Spring Festival vacation period started at least a week before this date time, leaving plenty of time for these people to leave the city. 

However, the number of people in Wuhan that travelled to reunite with their families in other cities during the Spring Festival vacation may be negligible, for the sealing-off of the city and other preventive measures taken across the country may have prevented their travelling. Second, the influence of the college students in Wuhan was an invariant factor, as college students are young and healthy, have fixed travelling routes, come from different regions evenly scattered across the country, and travelled to return home on or around January 10; we would assume their influence on virus transmission to different regions to be the same. Third, the medical treatment ability of regional medical centres of Hubei Province would also be the same, as the breakout emerged so fast that these regional medical centres would have had the same level of emergency-preparedness. Finally, the above factors will not change dramatically until the mass return of Wuhan’s floating population after the conclusion of the Spring Festival vacation.

## 4. Results

### 4.1. Description of Statistical Analysis

To estimate the floating population in the cities of Hubei Province and across the country, we must determine the floating population residing in Wuhan in 2019. As the statistics compiled by the Wuhan city government from 2019 have not been released, the data from previous years was used for this prediction. The prediction of floating population in Wuhan based on the statistics from previous years is presented in Table 3, demonstrating that there were approximately 2.43 million migrants living in Wuhan for more than six months in 2019.

However, if the predictions of the statistical data were combined with survey data, which was used in this study to estimate the origin of Wuhan’s floating population that return to their hometowns, there would have been a problem with inconsistent statistical strength. This would have resulted from the fact that the floating population measured by the government statistics department reflects those who have lived in Wuhan for more than 6 months, but the respondents in the survey have lived in Wuhan for over one month. A shorter defined residence time would have therefore produced a larger estimate of the population, and thus the total floating population in Wuhan, as determined from the CMDS data, was larger than the population as determined by the government statistics department.

On 22 January 2020, Xinhua News Agency (an official government media source) interviewed the mayor of Wuhan and reported that more than 5 million members of the floating population had returned to their hometowns before the Spring Festival holiday. This number stated (over 5 million) was more than twice the predicted value in this study (2.43 million), indicating that the statistical strength of the news report was based on a shorter period of residence, and this was consistent with the data we used to determine the floating population residing in Wuhan for over one month. Thus, in the absence of more rigorous and authoritative total data, we used 5 million people as Wuhan’s floating population, from which to estimate the scale and distribution of those members of this population who returned to their hometown during the Festival.

### 4.2. National Distribution and Social Characteristics of the Origins of Wuhan’s Floating Population

Based on sample survey data, in Table 4, the proportional estimation of the origins of Wuhan’s floating population at a provincial level is presented, as well as the results of statistical analysis based on a floating population of 5 million.

**Figure 1:**
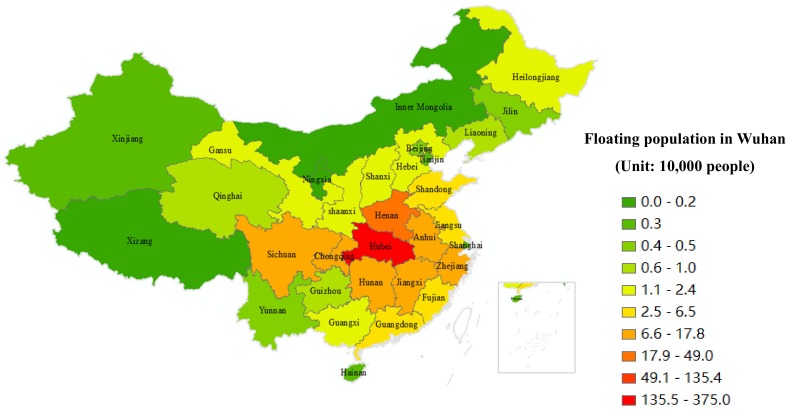
The national distribution of floating population in Wuhan.

The national distribution of the migrants presents obvious spatial characteristics of circle layers and echelons at provincial level (Table 4 and Figure 1).

(1) Hubei Province is the central area of origin of Wuhan’s floating population, accounting for 75% of the population, with a 95% confidence interval of (74.21, 75.76). Based on a total population of 5 million people, Wuhan’s floating population with household registration in Hubei Province is approximately 3.75 million, with a 95% confidence interval of (3,710,227 to 3,788,125).

(2) Henan, Anhui, Jiangxi and Hunan Provinces belong to the first circle layer. Henan Province, home to a floating population of 337,000, had the highest proportion with respect to its total population, equating to approximately 6.7% and a 95% confidence interval of (315,401 to 360,712).

Based on the analysis of city data in 2017, Xinyang, Zhumadian, Shangqiu, and Nanyang cities in Henan Province accounted for 38.82%, 20.59%, 12.94%, and 10.59% respectively, of the floating population from Henan living in Wuhan, accounting for approximately 83% of the total. The floating population proportions of Anhui, Hunan, and Jiangxi Provinces were 2.7%, 2.5%, and 2.4%, respectively, with corresponding floating populations in Wuhan of 136,000, 126,000, and 121,000 respectively.

(3) Chongqing, Zhejiang, Sichuan, Fujian and Jiangsu Provinces are at the second circle layer, with 1.65%, 1.39%, 1.39%, 1.00% and 0.92% floating populations, respectively, with corresponding populations of approximately 83,000, 70,000, 70,000, 50,000 and 46,000 respectively.

(4) Shandong, Guangdong, Hebei, Gansu, Guangxi, Heilongjiang, Shaanxi, Shanxi and Guizhou Provinces are at the third circle layer, with a proportion of 0.19% to 0.62% and a corresponding population of 10,000 to 30,000.

(5) Some provinces and municipalities, including Qinghai, Liaoning, Yunnan, Jilin and Beijing, are located in the fourth circle layer, accounting for 0.08–0.16% of the floating population, equating to 4000–8000 people.

(6) The remaining provinces and municipalities, such as Hainan, Xinjiang, Tianjin, Shanghai, Inner Mongolia, Tibet and Ningxia, are at the fifth circle layer, with a floating population proportion of less than 0.04%, corresponding to ≤2000 people.

As presented in the table above, this population is mainly 21–40 years old, but the scale of the susceptible, high-risk and over 40 years old population is also very large. The distribution is as follows:

(1) The susceptible and high-risk population is concentrated in Hubei Province. The size of the 41–50 age group is more than 800,000, that of the age group of 51–60 is 180,000, and the number of people over 60 is 40,000.

(2) Henan and Anhui Provinces have larger susceptible and high-risk populations, of more than 100,000 and nearly 40,000, respectively.

(3) Six provinces and municipalities, namely Hunan, Jiangxi, Chongqing, Zhejiang, Sichuan and Jiangsu, have a high-risk population of 41 to 50 years old, comprising 14,000–30,000 people.

(4) In 10 provinces, namely Fujian, Shandong, Guangdong, Hebei, Gansu, Guangxi, Shanxi, Guizhou, Qinghai and Xinjiang, the susceptible and high-risk populations are also concentrated in the 41–50 age group, with a population of approximately 1500–6550.

(5) The three provinces of northeast China, namely Heilongjiang, Jilin and Liaoning, have large susceptible and high-risk populations, equating to approximately 7000 in Heilongjiang and approximately 3000 in Jilin and Liaoning.

Infection of family members is a main means of transmission, and the distribution of the characteristics of floating population family migration at the provincial level are detailed in Table 5. The vast majority of the floating population migrates to Wuhan in the form of nuclear families (84.42%), and most families comprise 3–4 members (71.44%). The distribution is as follows:

(1) The number of nuclear family households in the Wuhan floating population that originates from Hubei Province is 3.425 million, accounting for 62.85% of the total floating population of Wuhan, and households with 3-4 family members number 2,693,500, accounting for 53.87% of the total. The high risk of 2019-nCoV transmission within and by this population is self-evident.

(2) Families from Henan, Anhui, Hunan and Jiangxi Provinces comprise a large proportion of those in the floating population of Wuhan. Those from Henan total nearly 300,000 households, and the number of these households with 3–4 family members is more than 240,000. Approximately 110,000 families from the remaining provinces are part of the floating population of Wuhan, including nearly 100,000 3–4 family-member households from Anhui and more than 80,000 from Hunan and Jiangxi.

(3) The number of families in the floating population of Wuhan from Chongqing, Zhejiang, Sichuan, Fujian and Jiangsu municipalities and provinces is 40,000–80,000, and the number of households with 3-4 family members is 30,000–60,000.

(4) The number of families in the floating population of Wuhan that originate from 7 other provinces, namely Shandong, Guangdong, Hebei, Gansu, Guangxi, Heilongjiang and Shaanxi, is 10,000–30,000 households, and the number of households with 3–4 family members is approximately 20,000. The remaining provinces comprise fewer than 10,000 households Jiangxi.

Certain factors can easily spread the virus from homes to communities in rural areas, such as a lack of medical resources and investment, weak health prevention and control, low awareness of health, and insufficient awareness of infectious diseases. In Table 5, the floating population in Wuhan is dominated by rural households (85.14%), and working or doing business is the main reason for their having travelled to Wuhan (84.29%). Therefore, epidemic prevention and control in rural areas is of critical importance. The distribution is as follows:

(1) The joint distribution of the origins of Wuhan’s floating population within Hubei Province is 63.73%, equating to a population of 3,186,500, and 62.71% of these are migrant workers, equating to 3,135,500 people.

(2) Henan, Anhui, Hunan, and Jiangxi Province both have more than 100,000 households with rural household registers and migrant workers in Wuhan, and the population of those from Henan in Wuhan’s floating population is approximately 300,000.

(3) Chongqing, Zhejiang, Sichuan, Fujian, Jiangsu, Shandong, Guangdong, Hebei, Gansu, and Guangxi Provinces together have a population of 10,000–80,000 households with rural household registers in Wuhan and less than 10,000 in the remaining provinces of China. Notably, Guangdong, Gansu, Heilongjiang and Liaoning have a larger proportion of the population with urban household registers, and this is greater than the number of rural household registers in Guangdong Province. 

Virus transmission is related to individual health awareness, which is affected by an individual’s educational level, so we also examined the educational level of the floating population in Wuhan. In Table 5, 60% of the population was educated to junior middle school and below, 26% had senior high school or technical secondary school education, and 14% had college education and above, indicating that the overall education level of this population was low. Specifically: 

(1) In the provinces of Qinghai, Chongqing, Jiangxi, Anhui, Henan, Yunnan, Guangxi and Xinjiang, 60% or more of the population was educated to junior high school level or below.

(2) Approximately 50–60 % of the population of the provinces of Hubei, Sichuan, Hebei, Fujian, Jiangsu, Hunan, Guizhou, Shandong, Shanxi, Tibet and Gansu was educated to junior high school level or below.

(3) The population in three municipalities, including Beijing, Tianjin and Shanghai, have a high level of education, with over 66% receiving tertiary education. The population of the remaining provinces had a medium-to-high educational level.

Above all, these data indicated that there is a large middle-aged and older high-risk floating population in Wuhan. Their social characteristics include having travelled to Wuhan in a nuclear family of 3–4 members, being on a rural household register, and often having a lower educational level. These characteristics are consistent with conditions favouring the wide spread of 2019-nCoV.

### 4.3. Distribution and Social Characteristics of the Origins of Wuhan’s Migrants in Hubei Province

According to the foregoing analysis, 75% of Wuhan’s floating population have registered households in Hubei Province, equating to approximately 3.75 million people. That such a large proportion of the floating population of Wuhan originate from elsewhere in Hubei Province has reduced the possibility of the epidemic spreading across the country, but all regions in Hubei Province are facing tremendous pressure from the spread of the epidemic. Therefore, we used the 2017 CMDS data to analyse the distribution of the floating population in regions within Hubei Province.

**Figure 2:**
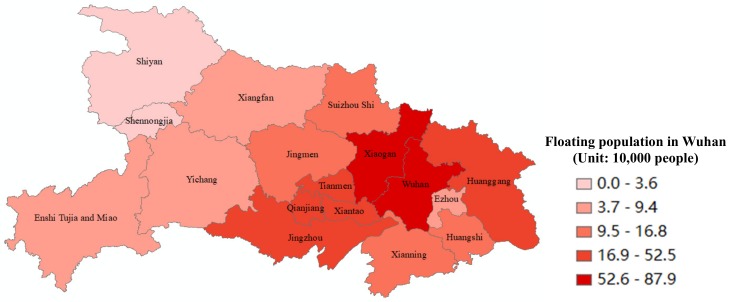
The distribution of Wuhan’s migrants within Hubei Province.

Table 6 and Figure 2 present the distribution of the origins of Wuhan’s floating population within Hubei Province. The proportion of the floating population gradually decreases from east to west across Hubei Province, and there are great differences between cities. The distribution is as follows:

(1) Xiaogan, Wuhan, and Huanggang are in the first echelon. The proportion of the floating population who originate from these cities is high, accounting for 23.4%, 19.6%, and 14% of the total, respectively. They are a cross-regional floating population of 734,000 and a 95% confidence interval of (65.89, 81.30). The analysis of districts and counties indicates that the members the floating population who originate from the outskirts of Huangpi District and Xinzhou District flow into the main urban area of Hankou, so the epidemic situation in the outskirts of Huangpi District and Xinzhou District needs special attention. Secondly, the members of Wuhan’s floating population who originate from Xiaogan comprise the largest proportion, equating to approximately 788,000 people and a 95% confidence interval of (79.81, 96.25). Members of Wuhan’s floating population who originate from Huanggang comprise the third proportion, equating to approximately 52,549 people and a 95% confidence interval of (46.06, 59.58). 

(2) The three Directly Managed by Province (DMP) cities (Xiantao, Qianjiang, and Tianmen) and Jingzhou belong to the second echelon, each comprising approximately 330,000 people, and each accounting for approximately 9% of the floating population of Wuhan, with a 95% confidence interval of (28,39).

(3) Jingmen, Suizhou, Xianning, and Huangshi belong to the third echelon, accounting for 3–5% of the floating population of Wuhan, equating to 130,000–170,000 people.

(4) Xiangyang, Ezhou, Yichang, Enshi, and Shiyan belong to the fourth echelon, accounting for less than 3% of the floating population of Wuhan, equating to fewer than 100,000 people.

Overall, the suburbs of Wuhan surrounding Xiaogan, Huanggang, and the three DMP cities are the origins of the largest proportion (66%) of the floating population of Wuhan, equating to approximately 2.475 million people.

We used district- and county-level variables to estimate the floating population within Hubei Province, and the results are presented in Table 7. The survey covered 94 districts and counties, including Huangpi, Xinzhou, Jiangxia, Caidian, and Hannan in Wuhan, as well as cross-region active migrants in some major urban areas.

The top 10 districts and counties of Hubei Province in terms of floating population are Huangpi, Hanchuan, Xiantao, Xinzhou, Hong’an, Yunmeng, Honghu, Macheng, Xiaonan, and Xiaochang. That is, ≥100,000 people from each of these districts and counties are part of the floating population of Wuhan, with the top 3 districts and counties, Huangpi, Hanchuan and Xiantao, having ≥200,000 people in Wuhan’s floating population.

These top 10 district and counties of Hubei Province are followed by Jingshan, Yingcheng, Dawu, Guangshui, Tianmen, Lishui, Jianli, Anlu, Jiangxia and Caidian, which each have 60,000–100,000 people in Wuhan’s floating population.

The third tier is Huangmei, Yangxin, Daye, Gongan, Tongshan, Jiayu, Zhongxiang, Qianjiang, Songzi, Huarong, Zengdu, Enshi, Liangzihu, Zaoyang, Dongxihu, Wuxue, Huangzhou, Hannan, Xian’an, Xiangzhou, Zhijiang, Echeng, Luotian, Badong, Chibi, Chongyang, Hongshan, Shayang, Shishou, Suixian, Tuanfeng, Gucheng and Xiangcheng. These districts and counties each have 10,000–50,000 people in Wuhan’s floating population.

The remaining districts and counties have fewer than 10,000 people in Wuhan’s floating population. 

In general, these members of Wuhan’s floating population originate from certain districts and counties of Hubei Province. The cumulative percentage of the top 30 districts and counties exceeds 80% of these areas’ total population, showing a clear exponential distribution trend.

We then analysed the social characteristics of the migrants in Hubei Province by age, type of migration, number of migrants, type of household registration, and reasons for traveling to Wuhan to become part of its floating population.

From Table 8 (please see the last page), we observe that in terms of susceptible and high-risk groups over 40 years old, there are approximately 300,000 people in Xiaogan, approximately 180,000 people in Wuhan (cross-region migration), and approximately 150,000 people in Huanggang. There are also approximately 100,000 people in the DMP Cities and Jingzhou respectively, and 30,000–50,000 people in Jingmen, Suizhou, Xianning, and Huangshi. Fewer than 30,000 people from each of Xiangyang, Ezhou, Yichang, Enshi and Shiyan have travelled to Wuhan.

The migration characteristics of the floating population of Wuhan from Hubei Province are detailed in Table 8. Migration with a nuclear family is the main pattern, accounting for nearly 80% of the total, or 2.985 million households. The proportion of households with 3–4 family members (i.e., nuclear families) is approximately 67%, or 2.53 million households. Specifically, 740,000 nuclear families originate from Xiaogan, 400,000–600,000 nuclear families originate from the inner suburbs of Wuhan and Huanggang, and approximately 260,000 nuclear families originate from the DMP cities and Jingzhou. More than 100,000 nuclear families originate from Jingmen, Suizhou, Xianning, and Huangshi, while fewer than 100,000 nuclear families originate from Xiangyang, Ezhou, Yichang, Enshi and Shiyan. The distribution of households with 3-4 members is similar to that of nuclear families.

It also presents the distribution of the origins of Wuhan’s floating population who originate from within Hubei Province. According to the statistical results, rural household registers account for 83%, equating to a population of approximately 3.12 million. The proportion of the group who was working and doing business in urban areas is 77%, and the population is 2.89 million. The size of the population distribution in each city is similar to the aforementioned migration types and other variables, and is not reported here.

In Table 8, the overall educational level of those members of Wuhan’s floating population who originate from Hubei Province is higher than the national level, with approximately 52% having been educated to junior high school level and below, approximately 29% to high school/secondary school level and below, and approximately 19% to college and above. However, in those members of Wuhan’s floating population who originate from the surrounding cities of Wuhan, which contribute a large number of people to the floating population of Wuhan, namely Xiaogan, Huanggang, Huangshi, Suizhou, DMP cities, Xianning, and Ezhou, >50% of people have an educational level of junior high school and below, with this being >60% in Xiaogan. This means that the awareness of health protection and timely treatment may be low in this section of the floating population of Wuhan, which will heighten the risk of large-scale transmission of 2019-nCoV.

### 4.4. Prediction of Epidemic Trends within Hubei Province 

The floating population in Wuhan will serve as a sound predictor for the trend of the 2019-nCoV outbreak. The Pearson’s correlation coefficient between the proportion of the floating population in Wuhan who originate from a certain region of Hubei and the number of confirmed 2019-nCoV cases in each region increased from 0.65 on 25 January 2020 to 0.84 on 31 January 2020 (Table 9). This indicates that when a region contributes a higher number of people to the floating residential population of Wuhan, more confirmed cases will emerge in this region. 

**Figure 3:**
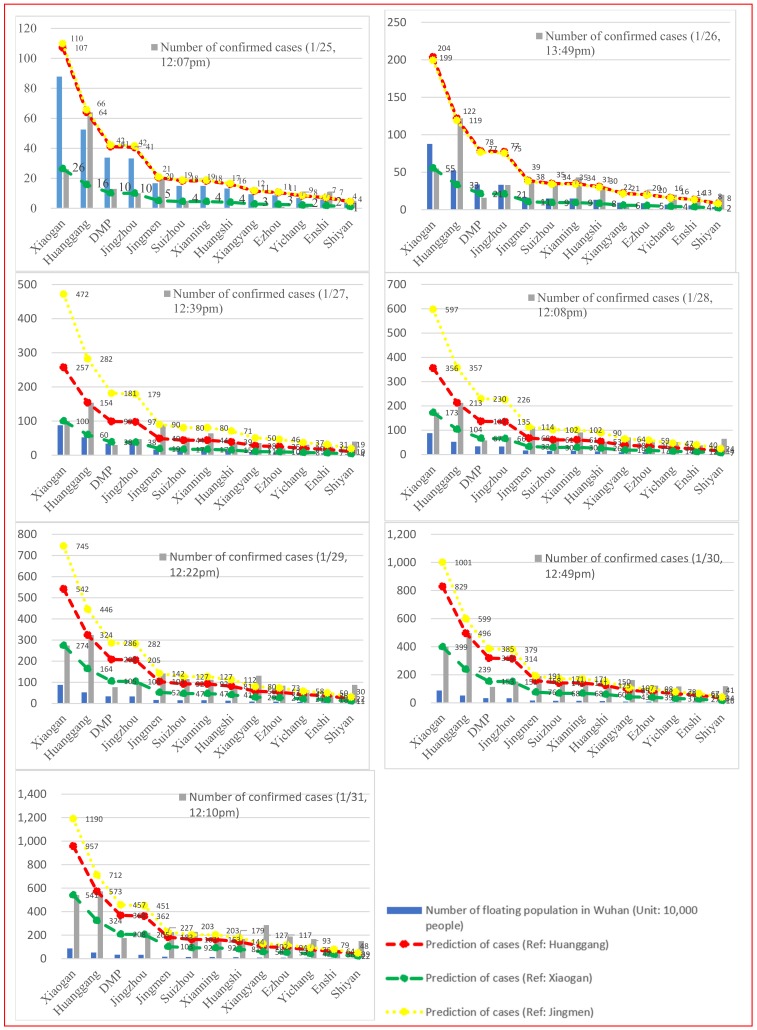
Prediction of the epidemic situation in Hubei Province.

We assumed that the effect of the floating population on the transmission of the 2019-nCoV is consistent across Hubei province, and selected three prefectures that contribute the greatest number of people to the floating population of Wuhan (Xiaogan, Huanggang and Jingmeng) as the reference prefectures to predict the epidemic trend of the 2019-nCoV at prefecture level. Those prefectures can be divided into three groups since 28 January 2020 (Figure 3): (1) Prefectures with a rapid increase in confirmed cases, comprising Jingmen, Xianning, Xiangyang, Ezhou, Yichang, and Enshi; (2) Prefectures with a moderate growth in confirmed cases, comprising Huanggang, Suizhou, Huangshi, and Shennongjia; and (3) Prefectures with a slow increase in confirmed cases, comprising Xiaogan, DMP cities, and Jingzhou. 

Our predictions of the epidemic trends of the 2019-nCoV outbreak within Hubei Province are as follows: (1) The epidemic growth model for Jingmen predicts a rapid increase in the number of confirmed cases in Xiaogan, DMP cities, and Jingzhou. In other words, if the epidemic pattern in Jingmen is regarded as a typical evolutionary pattern of the spread of 2019-nCoV, the actual number of confirmed cases of 2019-nCoV in Xiaogan, DMP cities, and Jingzhou have been greatly underestimated. For example, the number of confirmed cases in Xiaogan on 31 January 2020 would reach 1190, but the number of officially announced cases was only 541. This could be attributable to a lack of sufficient diagnostic capabilities, or undisclosed data from the government, or both. (2) The epidemic growth model for Xiaogan predicts a rapid increase in the number of confirmed 2019-nCoV cases in Jingmen, Xianning, Xiangyang, Ezhou, Yichang and Enshi. If this is true, large-scale outbreaks have occurred in these places, but their regional governments have either not detected this, or true figures are not available or revealed. (3) The epidemic growth model for Huanggang better fits the actual epidemic situation of the 2019-nCoV outbreak, based on the floating population and the intensity of medical intervention. The outbreak in Xiaogan, DMP cities, and Jingzhou may be underestimated, while the rapid increase in the number of confirmed cases in Jingmen, Xianning, and Xiangyang may be affected by other unknown factors or uncontrollable random factors that need further investigation.

### 4.5. Prediction of Epidemic Trends outside Hubei Provinces

The floating population of Wuhan originated from outside Hubei Province may have promoted the spread of 2019-nCoV. Table 10 compares the number of individuals travelling from Wuhan to other provinces and the daily number of confirmed cases for those other provinces. Analysis revealed that the correlation coefficient at the provincial level was lower than at the prefecture level within Hubei Province, but the correlation coefficient increased from 0.4 on 25 January 2020 to 0.63 on 31 January 2020.

**Figure 4:**
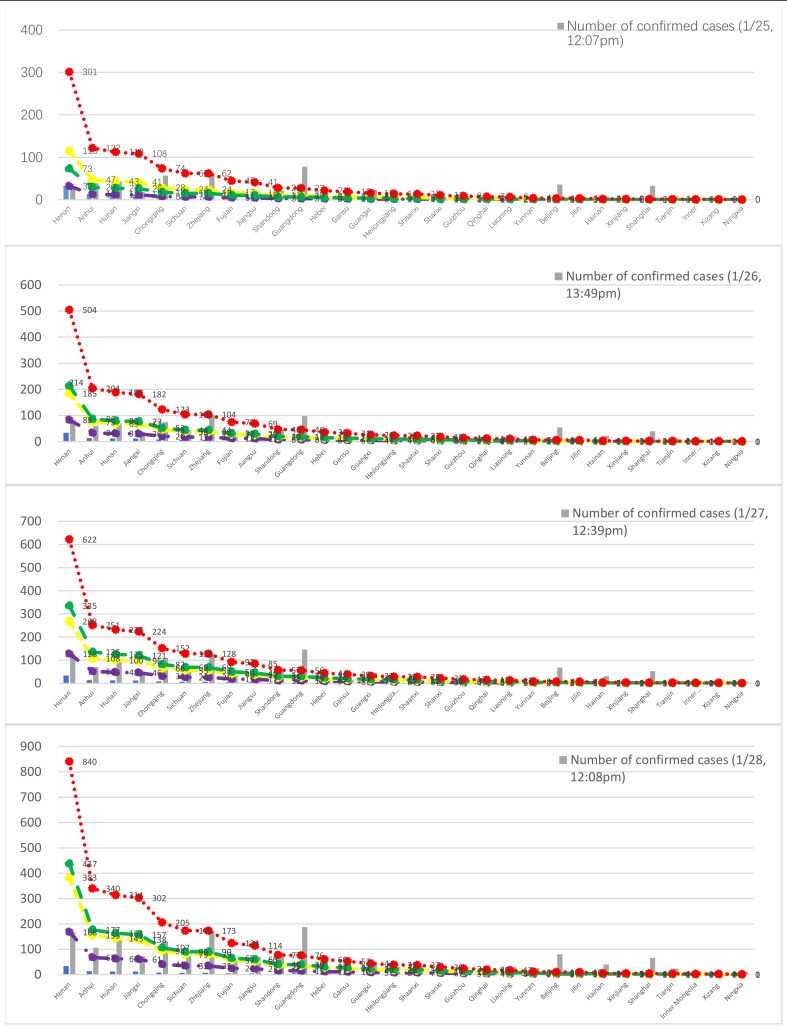

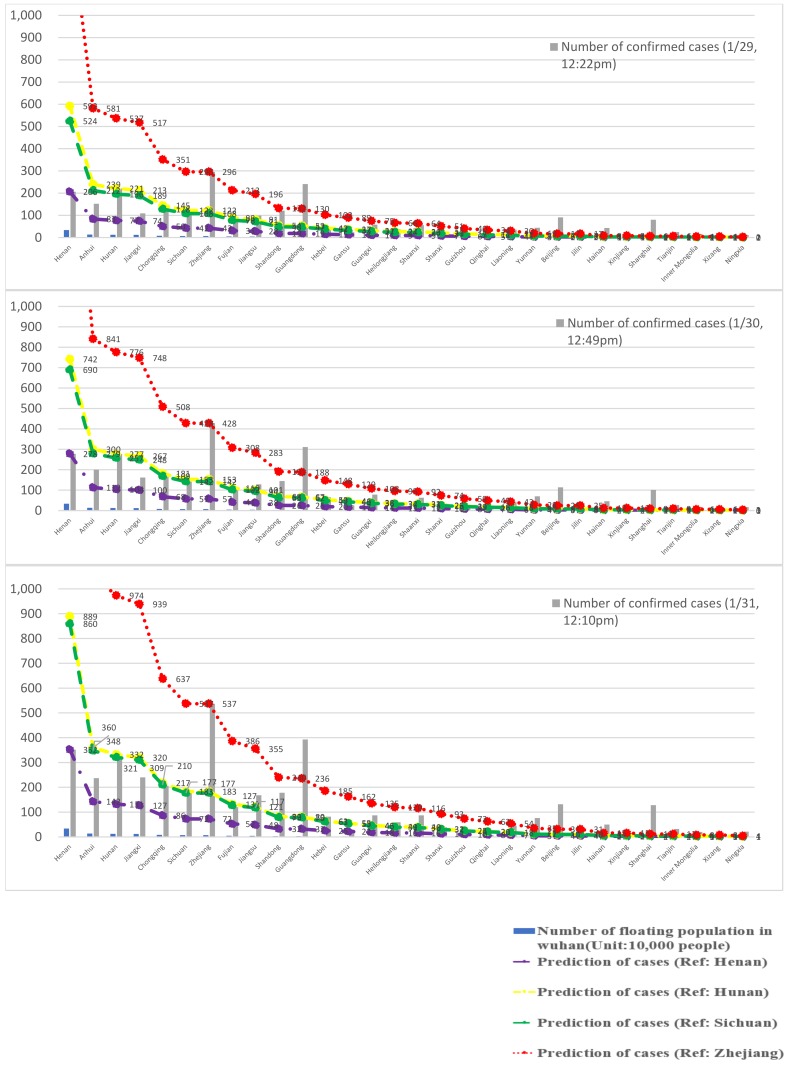
Prediction of the epidemic situation in China.

Table 10 also shows the ratio of confirmed cases in each province to the proportion of people in the floating population in Wuhan who originate from each of these provinces, on 28 January 2020. We divide provinces into two categories based on their short-term travel populations in Wuhan, and Wuhan’s travelling population to other provinces during the Spring Festival holiday. The first category comprises those provinces that have large-scale short-term business trips or tourist populations in Wuhan during the Spring Festival holiday, namely Beijing, Shanghai, Tianjin, and Hainan. Obviously, such a high level of inter-provincial population mobility may exacerbate the spread of 2019-nCoV. For example, the high ratio of confirmed cases in Guangdong Province may be due to the large short-term travel populations visiting Shenzhen and Guangzhou and Wuhan, while the high ratio of confirmed cases in Hainan Province may result from the outbound tourist population from Wuhan to Hainan during the Spring Festival holiday. In Table 10, the results are divided into two parts: the correlation coefficient of the first category of provinces, which reaches a maximum of 0.96, and the correlation coefficient of the second category of provinces, which increased from 0.56 to 0.7. This abovementioned second category comprise the other 25 provinces that have small short-term business trip groups or tourist populations in Wuhan during the Spring Festival holiday. We assumed that the effect of the floating population on the spread of 2019-nCoV was consistent across the country. The other 25 provinces are divided into three groups since 25 January 2020 (Figure 4): (1) Provinces with a rapid increase in the number of confirmed cases, namely Zhejiang, Shandong, Guangxi, Shaanxi, Liaoning, and Yunnan; (2) Provinces with a moderate increase in the number of confirmed cases, namely Hunan, Chongqing, Sichuan, Fujian, Jiangsu, Hebei, Gansu, Heilongjiang, Shaanxi, Guizhou, Qinghai, Jilin, Xinjiang, Inner Mongolia, Tibet, and Ningxia; and (3) Provinces with a small increase in the number of confirmed cases, namely Henan, Anhui, and Jiangxi. In Table 10, if we exclude the data of Henan Province and Zhejiang Province from the second category, we find that the correlation coefficient on 31 January 2020 is 0.93.

We selected four provinces (Henan, Hunan, Sichuan, and Zhejiang) as the reference provinces to predict the epidemic trend of 2019-nCoV in each province. We found that: (1) The epidemic growth model of Henan Province does not fit the situation in most other provinces. That is, except in Anhui and Jiangxi, the actual number of outbreaks in other provinces was higher than that predicted by the Henan model. As these provinces have large floating populations in Wuhan, the rapid increase in the number of confirmed cases in Henan, Anhui and Jiangxi may result from effective measures that have been taken to control the spread of 2019-nCoV, or the lack of sufficient diagnostic capabilities to detect suspected cases. (2) The epidemic growth model for Hunan and Sichuan Province predicts a rapid increase in the number of confirmed cases in Henan, Anhui and Jiangxi provinces. Thus, if the epidemic pattern in Hunan and Sichuan follows a typical evolutionary pattern, the current numbers of confirmed cases in the three provinces of Henan, Anhui, and Jiangxi are greatly underestimated. For example, the number of confirmed cases in Henan on 31 January 2020 would be between 860 and 889, but the number in official announcements was only 168. In contrast, the number of confirmed cases in Zhejiang, Shandong, Guangxi, Shaanxi, Liaoning, and Yunnan Provinces were higher than the predicted number, which may be affected by uncontrollable local factors that need further investigation. (3) The epidemic growth model for Zhejiang Province predicts a rapid increase in the number of confirmed cases in most provinces, especially Jiangsu and Fujian provinces that are adjacent to Zhejiang. It is important to investigate why there were so many confirmed cases in Zhejiang, and whether the outbreak in Jiangsu and Fujian Province was not detected in a timely manner, or whether all possible cases have not yet occurred.

Overall, the predicted epidemic pattern for Hunan and Sichuan provinces fits best to the actual epidemic trend of the 2019-nCoV outbreak. However, the current number of confirmed cases in Henan, Anhui, and Jiangxi provinces is likely to be underestimated, especially given that these contain extensive rural areas with large populations and limited medical resources. The higher actual number of confirmed cases in Zhejiang, Shandong, Guangxi, Shaanxi, Liaoning, and Yunnan provinces may be affected by other unknown factors or uncontrollable random factors that need further investigation.

## 5. Discussion

To prevent or mitigate the spread of an emerging infectious disease and its negative effects, public health interventions mainly aim at three types of population, namely the population in the source area, the floating population leaving the source area, and the population travelling from the infected area to other areas. The Spring Festival in 2020 is much earlier than in previous years. At this time, the possibility of human-to-human transmission of a new coronavirus had just been discovered. When the Wuhan Municipal Government decided on 23 January 2020 to “close the city” to control the outflow of population, more than 5 million people had already left Wuhan on the Spring Festival holiday, and it was too late to control the entire potentially infected population in the epidemic area. At present, China’s high-speed railway and expressway transportation network has experienced great development. This fast and convenient transportation has led to a floating population that can leave the source area to quickly reach every part of the country, which makes it very difficult to quarantine the floating population leaving the source area through transportation stations. In addition, there is an incubation period after human infection, further increasing the difficulty of quarantine at traffic stations, which is also an important reason for the implementation of “city closure” control policies in many cities across the country.

After 2019-nCoV was confirmed as being capable of transmitting from human to human, the Chinese government implemented top to bottom national mobilisation. It fully investigated and isolated the population of Wuhan, and also publicised the severity of the epidemic, and also increased awareness of the prevention of infectious diseases and raised people’s vigilance through messages on television, mobile communications and the Internet. In addition, according to the latest epidemic surveillance, the incubation period of the coronavirus is 3 to 7 days, with an upper limit of 14 days. For this reason, the central government has issued an executive order to extend the Spring Festival holiday from 30 January to 2 February 2020. Many provinces are even requiring firms to not restart work until 9 February, except those necessary for social operations related to the national economy and people’s livelihood. Extending the holiday is needed to avoid the returning people leaving home early and returning to work, so as to minimise the risk of the epidemic spreading again due to population fluctuations.

There are limitations to this study. First, our analysis did not include other large-scale populations. For example, some are college students, because Wuhan is the city with the largest number (>1 million) of college students in China and the world. The other parts include short-term business travellers, transit passengers and tourists. Official media reported that the size of the populations during the Spring Festival holiday would reach more than 30 million. This can be confirmed from the daily-confirmed cases of 2019-nCoV infection. Although there is a small permanent population in Wuhan whose household register belongs to provinces and cities such as Beijing, Shanghai, Tianjin, Hainan, and Guangdong (in fact, Shenzhen and Guangzhou are two megacities), these provinces and cities still have large-scale temporary floating populations from and to Wuhan because of the large population and well-developed economy. Therefore, the number of confirmed cases of 2019-nCoV infection in these areas is far ahead of that in most other provinces that have a large floating population in Wuhan. 

Second, our sample has a certain deviation. The data on the origin of Wuhan’s floating population does not include Hong Kong, Macao, or international migrants, which makes our research unable to estimate the population size of these regions. At present, some cases have been confirmed in surrounding Asian countries, Europe, North America and Australia. Third, limited to interdisciplinary research capabilities, our model does not include infectious disease analysis models such as SIR to further analyse the potential and scale of 2019-nCoV spread, which may reduce the value of this research in the prevention and control of 2019-nCoV infections. Finally, the results of the study are mainly applicable to the end of the Spring Festival holiday, and after the large-scale population comes back to work or study, the spread of the epidemic will be more complicated.

We believe that the abovementioned limitations can be overcome. Using big data such as location information of transportation and mobile Internet, short-term floating populations can be included in the study to maximise the estimated population flotation and scale in Wuhan. Unfortunately, thus far we have not seen a rigorous study using big data to analyse the outflow of populations in the epicentre of an epidemic. This means that there is still a long way to go for the research and application of big data in the field of national and global public health.

## 6. Conclusions

At the time of writing this paper (29 January 2020), all provinces in China have reported confirmed or suspected cases of 2019-nCoV, every prefecture and city in Hubei Province has confirmed cases of 2019-nCoV, and transmission of 2019-nCoV has spread from imported to inter-regional. Due to the fact that 5 million migrants had left Wuhan before the “closure of the city”, our research reveals a high correlation between the number of Wuhan’s floating population and the number of confirmed cases. Fortunately, the origin of Wuhan’s floating population is highly concentrated in Hubei Province and its surrounding provinces, of which the migrants with Hubei household registers account for 75%, and more than 80% of the population is concentrated in the top 30 districts and counties. This means that some areas will face a very high risk of epidemic outbreaks, but it is also conducive to centralised resources enabling prevention and control of the epidemic to avoid large-scale spread in other regions.

More than 5 million of Wuhan’s floating population have returned to their hometowns as potential carriers of the virus and may become carriers of the virus’s re-transmission. Due to China’s urban and rural dualistic structure, most of these people are rural migrant workers with low levels of education. The results find that 85% of the migrants have rural household registers. These people, who frequently work outdoors or work overtime are more likely to be susceptible because of their poor diet and nutrition. At the same time, most of these people travel with 3–4 family members, and the susceptible and high-risk population over 40 years old accounts for a large proportion of this floating population, which provides ideal conditions for the transmission of 2019-nCoV within families. To make matters worse, the rural areas where these people return to have very limited medical and public health services, and gatherings during the Spring Festival aggravate the risk of virus transmission in the community.

So far, confirmed cases of 2019-nCoV continue to increase every day across China. The results of our model analysis indicate that, on the one hand, the correlation between the size of the floating population and the number of confirmed cases in Wuhan has continued to increase over time, and by 28 January, the correlation coefficient of these factors in Hubei Province had reached 0.78, which means that the size of the floating population in Wuhan is an important parameter for predicting the epidemic. On the other hand, we also found that the effect of the size of the floating population in Wuhan is heterogeneous across regions. Some areas have a large floating population in Wuhan, including Henan, Anhui, and Jiangxi provinces, and Xiaogan City, Jingzhou City, and the three county-level cities directly under the provincial government, and yet the number of confirmed cases of 2019-nCoV is apparently relatively small. However, we believe that the epidemic situation in these areas may be underestimated. Considering the serious consequences of delays in diagnosis and loopholes in infection control in suspected or confirmed cases of SARS in the SARS epidemic in 2003, it is necessary to strengthen surveillance in these areas to determine the causes of the fewer confirmed cases of 2019-nCoV in these areas.

## Figures and Tables

**Table 1:** Sample distribution of floating population living in Wuhan, 2013–2018.

District	2013	2014	2015	2016	2017	2018	Total
Total	1999	2000	2000	2000	2000	2000	11,999
Hankou zone							
Jiang’an	400	360	360	320	400	-	1840
Qiaokou	240	240	240	200	240	-	1160
Jianghan	280	240	200	200	160	-	1080
Dongxihu	200	160	200	320	200	-	1080
Huangpi	120	120	120	120	160	-	640
Xinzhou	0	0	40	40	0	-	80
Wuchang zone							
Hongshan	239	240	240	240	200	-	1159
Wuchang	200	160	200	200	240	-	1000
Qingshan	80	160	120	80	40	-	480
Jiangxia	40	0	40	40	40	-	160
Hanyang zone							
Hanyang	120	160	160	160	200	-	800
Hannan	40	120	80	80	80	-	400
Caidian	40	40	0	0	40	-	120
All three zones	-	-	-	-	-	2000	2000

**Table 2:** Household registration distribution of floating population in Wuhan, 2013–2018.

Province	2013	2014	2015	2016	2017	2018	Total
Total	1999	2000	2000	2000	2000	2000	11,999
Hubei	1514	1508	1487	1465	1477	1547	8998
Henan	113	134	109	159	170	125	810
Anhui	59	58	55	53	56	46	327
Hunan	57	46	68	54	41	36	302
Jiangxi	58	40	53	57	49	34	291
Chongqing	34	29	34	33	33	35	198
Zhejiang	22	29	25	33	25	33	167
Sichuan	22	30	45	21	22	27	167
Fujian	14	17	16	15	39	19	120
Jiangsu	38	13	16	19	13	11	110
Shandong	12	18	11	13	8	12	74
Guangdong	7	8	18	18	14	8	73
Hebei	9	15	3	9	10	12	58
Gansu	4	14	9	6	7	10	50
Guangxi	9	8	8	7	3	7	42
Heilongjiang	5	7	6	7	7	5	37
Shaanxi	8	2	8	5	6	7	36
Shanxi	2	3	3	8	4	9	29
Guizhou	2	5	5	4	4	3	23
Qinghai	5	3	6	2	0	3	19
Liaoning	2	2	1	6	5	1	17
Yunnan	0	1	5	3	0	2	11
Jilin	0	4	1	0	3	2	10
Beijing	1	2	3	1	2	0	9
Hainan	0	0	2	0	1	2	5
Xinjiang	0	3	1	0	0	1	5
Tianjin	1	0	0	1	0	1	3
Shanghai	0	1	0	1	0	1	3
Inner Mongolia	1	0	0	0	1	0	2
Xizang	0	0	1	0	0	1	2
Ningxia	0	0	1	0	0	0	1

**Table 3:** Prediction of floating population living in Wuhan, 2019.

Population Type	2013	2014	2015	2016	2017	2018	2019
Permanent population at the year-end	10,220,000	10,338,000	10,607,700	10,766,200	10,892,900	11,081,000	11,263,800
Household population	8,220,500	8,273,100	8,292,700	8,338,400	-	8,837,300	8,896,900
Floating population (calculate) ^1^	1,999,500	2,064,900	2,315,000	2,427,800	-	2,243,700	2,423,700
Floating population (predicted)	2,078,700	2,138,400	2,198,200	2,258,000	2,317,800	2,377,600	2,437,400

From 2013 to 2018, the values are from Wuhan Statistical Bulletin of the National Economic and Social Development. In 2019, the values are based on linear regression; ^1^ Floating population (calculate) = Permanent population at the year-end − Household population. Predicted value based on linear regression.

**Table 4:** Estimation of proportion and population size in China based on floating population in Wuhan, 2013–2018.

Province	Population Size in Wuhan	Percentage (95% CI)	Estimation of Population Size (95% CI)
Total	11,999	100	5,000,000
Hubei	8998	75.0 (74.2–75.8)	3,749,479 (3,710,227 – 3,788,125)
Henan	810	6.8 (6.3–7.2)	337,528 (315,401–360,712)
Anhui	327	2.7 (2.4–3.0)	136,262 (122,063–151,622)
Hunan	302	2.5 (2.2–2.8)	125,844 (112,201–140,665)
Jiangxi	291	2.4 (2.2–2.7)	121,260 (107,869–135,822)
Chongqing	198	1.7 (1.4–1.9)	82,507 (71,491–94,717)
Zhejiang	167	1.4 (1.2–1.6)	69,589 (59,493–80,888)
Sichuan	167	1.4 (1.2–1.6)	69,589 (59,493–80,888)
Fujian	120	1.0 (0.8–1.2)	50,004 (41,492–59,734)
Jiangsu	110	0.9 (0.8–1.1)	45,837 (37,702–55,194)
Shandong	74	0.6 (0.5–0.8)	30,836 (24,228–38,681)
Guangdong	73	0.6 (0.5–0.8)	30,419 (23,859–38,218)
Hebei	58	0.5 (0.4–0.6)	24,169 (18,362–31,222)
Gansu	50	0.4 (0.3–0.5)	20,835 (15,472–27,450)
Guangxi	42	0.4 (0.3–0.5)	17,502 (12,619–23,643)
Heilongjiang	37	0.3 (0.2–0.4)	15,418 (10,860–21,239)
Shaanxi	36	0.3 (0.2–0.4)	15,002 (10,511–20,756)
Shanxi	29	0.2 (0.2–0.3)	12,085 (8096–17,346)
Guizhou	23	0.2 (0.1–0.3)	9584 (6078–14,374)
Qinghai	19	0.2 (0.1–0.2)	7918 (4768–12,359)
Liaoning	17	0.1 (0.1–0.2)	7084 (4128–11,337)
Yunnan	11	0.1 (0.05–0.2)	4584 (2289–8199)
Jilin	10	0.1 (0.04–0.2)	4167 (1999–7661)
Beijing	9	0.1 (0.03–0.14)	3751 (1715–7117)
Henan	5	0.04 (0.01–0.1)	2084 (677–4861)
Xinjiang	5	0.04 (0.01–0.1)	2084 (677–4861)
Tianjin	3	0.03 (0.01–0.1)	1250 (258–3653)
Shanghai	3	0.03 (0.01–0.1)	1250 (258–3653)
Inner Mongolia	2	0.02 (0.002–0.1)	834 (101–3010)
Xizang	2	0.02 (0.002–0.1)	834 (101–3010)
Ningxia	1	0.008 (0.0002–0.05)	417 (11–2322)

Estimation of population size is based on the total number of floating population in Wuhan (about 5 million); CI = confidence interval.

**Table 5:** Joint distribution of Wuhan floating population in China.

Province	Total	Age Group						Migration Characteristics			Number of Migrant Family Members					Household Registration		Reason for Migration			Education Level		
		<20	21–30	31–40	41–50	51–60	60+	Migration Alone	Nuclear Family Migration	Extended Family Migration	1	2	3	4	≥5	Rural	Non-Rural	Work, Business	With Family	Other Reason	Junior High School And Below	High School	College and Above
Total	5,000,000	158,763	1,740,145	1,730,561	1,094,675	227,936	47,921	483,790	4,213,268	302,942	483,790	750,479	2,345,195	1,226,769	193,766	4,255,355	742,562	4,214,518	599,217	186,266	3,007,334	1,308,442	684,224
Hubei	3,749,479	112,093	1,283,440	1,307,192	824,652	180,848	41,253	377,948	3,142,345	229,186	377,948	539,628	1,800,567	892,991	138,345	3,185,265	564,214	3,135,678	456,705	3,135,678	2,224,352	1,006,334	518,793
Henan	337,528	12,918	123,344	101,258	85,007	13,751	1250	24,585	292,941	20,002	24,585	50,004	137,511	104,175	21,252	310,443	27,086	294,191	34,586	294,191	236,686	70,423	30,419
Anhui	136,261	5000	47,504	45,837	31,253	5417	1250	9167	118,343	8751	9167	27,919	52,088	40,420	6667	124,594	11,668	119,593	15,001	119,593	96,258	26,669	13,334
Hunan	125,844	3750	47,921	45,420	25,002	3750	0	13,334	105,842	6667	13,334	25,002	56,255	27,502	3750	107,092	18,752	107,092	16,668	107,092	72,089	37,920	15835
Jiangxi	121,260	5834	49,171	41,253	20,835	4167	0	12,501	100,008	8751	12,501	20,002	43,754	37,086	7917	101,675	19,585	103,759	14,168	103,759	85,840	25,002	10,418
Chongqing	82,507	4584	22,919	26,252	23,752	4584	417	5417	71,256	5834	5417	18,752	33,753	21,668	2917	73,339	9167	70,839	6667	70,839	61,255	15,835	5417
Zhejiang	69,589	1667	19,585	24,585	18,335	4584	833	2917	64,589	2084	2917	10,001	35,836	17,918	2917	57,505	12,084	62,505	6251	62,505	33,336	22,919	13334
Sichuan	69,589	2500	27,086	22,085	14,585	2917	417	5834	58,755	5000	5834	12,501	32,919	16,668	1667	61,672	7917	56,671	10,834	56,671	40,837	18,752	10001
Fujian	50,004	2084	18,335	22,502	6251	833	0	5417	42,504	2084	5417	5834	22,085	15,001	1667	43,337	6667	46,254	3334	46,254	29,169	14,585	6251
Jiangsu	45,837	2500	14,585	14,168	12,501	1667	417	417	42,087	3334	417	8334	24,585	11,668	833	40,003	5834	39,170	6251	39,170	26,669	13,751	5417
Shandong	30,836	417	12,501	12,501	4584	833	0	3334	24,585	2917	3334	5834	12,918	8334	417	25,419	5417	26,669	3750	26,669	16,251	7917	6667
Guangdong	30,419	417	12,918	12,918	3334	833	0	3750	25,002	1667	3750	5417	15,001	6251	0	14,585	15,835	24,585	5000	24,585	8751	9584	12,084
Hebei	24,169	0	10,001	8751	4584	417	417	1250	22,502	417	1250	2084	10,834	8751	1250	21,252	2917	21,252	2500	21,252	14,168	7917	2084
Gansu	20,835	1667	9167	7917	2084	0	0	2084	17,918	833	2084	4167	11,668	1250	1667	15,001	5834	16,668	3750	16,668	10,001	4167	6667
Guangxi	17,501	417	7917	7501	1250	417	0	2917	14,585	0	2917	417	10,834	3334	0	15,001	2500	13,334	3334	13,334	10,834	4167	2500
Heilongjiang	15,418	0	4167	4584	5000	833	833	417	13,751	1250	417	3750	10,418	833	0	9167	6251	13,751	1250	13,751	6251	6667	2500
Shaanxi	15,001	0	5834	8334	417	417	0	3334	10,001	1667	3334	833	8751	833	1250	11,251	3750	13,751	1250	13,751	5417	4167	5417
Shanxi	12,084	417	4584	5417	1667	0	0	1667	9167	1250	1667	1250	4167	4584	417	9584	2500	10,834	833	10,834	6251	2917	2917
Guizhou	9584	417	5000	2500	1667	0	0	833	8751	0	833	1250	5417	2084	0	7501	2084	7084	2500	7084	5417	2084	2084
Qinghai	7917	417	3750	2084	1667	0	0	0	7501	417	0	1667	3750	2084	417	7917	0	7084	833	7084	7501	417	0
Liaoning	7084	0	1667	2917	1250	1250	0	1667	5000	417	1667	1667	3334	417	0	2500	4584	6251	417	6251	1667	1667	3750
Yunnan	4584	833	2917	833	0	0	0	417	3750	417	417	833	2084	833	417	4167	417	3334	1250	3334	2917	417	1250
Beijing	4167	0	417	833	2917	0	0	833	3334	0	833	1667	1250	417	0	1250	2917	4167	0	4167	1250	1667	1250
Jilin	3750	417	1250	1250	833	0	0	1667	2084	0	1667	0	2084	0	0	833	2917	3334	417	3334	1250	0	2500
Hainan	2084	0	1250	417	0	417	0	833	1250	0	833	0	1250	0	0	833	1250	1667	417	1667	417	833	833
Xinjiang	2084	0	833	0	1250	0	0	417	1667	0	417	0	417	1250	0	2084	0	1667	417	1667	1250	417	417
Tianjin	1250	417	417	417	0	0	0	417	833	0	417	417	0	417	0	833	417	833	417	833	417	0	833
Shanghai	1250	0	833	0	0	0	417	417	833	0	417	833	0	0	0	0	1250	1250	0	1250	0	417	833
Inner Mongolia	833	0	417	417	0	0	0	0	833	0	0	0	833	0	0	417	417	417	417	417	0	417	417
Xizang	833	0	0	417	0	0	417	0	833	0	0	417	417	0	0	417	417	417	0	417	417	417	0
Ningxia	417	0	417	0	0	0	0	0	417	0	0	0	417	0	0	417	0	417	0	417	417	0	0

**Table 6:** Estimation of proportion and population size in Hubei Province based on floating population in Wuhan, 2017.

City	Population Size in Wuhan	Percentage (95% CI)	Estimation of Population Size (95% CI)
Total	1477	100	3,749,479
Xiaogan	346	23.4 (21.3–25.7)	878,348 (798,137–962,540)
Wuhan	289	19.6 (17.6–21.7)	733,649 (658,849–813,015)
Huanggang	207	14.0 (12.3–15.9)	525,486 (460,570–595,817)
DMP cities ^1^	133	9.0 (7.6–10.6)	337,631 (284724–396,739)
Jingzhou	131	8.9 (7.5–10.4)	332,554 (280,029–391,301)
Jingmen	66	4.5 (3.5–5.7)	167,546 (130,201–211,854)
Suizhou	59	4.0 (3.1–5.1)	149,776 (114,529–192,075)
Xianning	59	4.0 (3.1–5.1)	149,776 (114,529–192,075)
Huangshi	52	3.5 (2.6–4.6)	132,006 (98,999–172,155)
Xiangyang	37	2.5 (1.8–3.4)	93,927 (66,358–128,851)
Ezhou	34	2.3 (1.6–3.2)	86,312 (59,966–120,059)
Yichang	27	1.8 (1.2–2.6)	68,542 (45,296–99,309)
Enshi	23	1.6 (1.0–2.3)	58,387 (37,106–87,268)
Shiyan	14	0.9 (0.5–1.6)	35,540 (19,465–59,439)

^1^ DMP (Directly Managed by the Province) cities includes Xiantao, Qianjiang and Tianmen; CI = confidence interval.

**Table 7:** Estimation of proportion and population size within Hubei Province based on floating population in Wuhan, 2017.

No.	District or County	Population Size in Wuhan	Percentage (95% CI)	Estimation of Population Size (95% CI)
Total		1477	100	3,749,476
1	Huangpi	139	9.4 (8.0–11.0)	352,862 (298,830–413,031)
2	Hanchuan	95	6.4 (5.2–7.8)	241,165 (196,273–292,679)
3	Xiantao	82	5.6 (4.4–6.8)	208,163 (166,459–256,639)
4	Xinzhou	66	4.5 (3.5–5.7)	167,546 (130,201–211,854)
5	Hong’an	60	4.1 (3.1–5.2)	152,315 (116,759–194,908)
6	Yunmeng	49	3.3 (2.5–4.4)	124,390 (92,395–163,568)
7	Honghu	46	3.1 (2.3–4.1)	116,775 (85,825–154,948)
8	Macheng	46	3.1 (2.3–4.1)	116,775 (85,825–154,948)
9	Xiaonan	42	2.8 (2.1–3.8)	106,620 (77,125–143,397)
10	Xiaochang	41	2.8 (2.0–3.7)	104,082 (74,961–140,497)
11	Jingshan	39	2.6 (1.9–3.6)	99,005 (70,649–134,685)
12	Yingcheng	39	2.6 (1.9–3.6)	99,005 (70,649–134,685)
13	Dawu	38	2.6 (1.8–3.5)	96,466 (68,501–131,771)
14	Guangshui	38	2.6 (1.8–3.5)	96,466 (68,501–131,771)
15	Tianmen	35	2.4 (1.7–3.3)	88,850 (62,091–122,996)
16	Xishui	35	2.4 (1.7–3.3)	88,850 (62,091–122,996)
17	Jianli	34	2.3 (1.6–3.2)	86,312 (59,966–120,059)
18	Anlu	32	2.2 (1.5–3.0)	81,234 (55,737–114,166)
19	Jiangxia	29	2.0 (1.3–2.8)	73,619 (49,448–105,275)
20	Caidian	28	1.9 (1.3–2.7)	71,080 (47,368–102,296)
21	ND ^1^	26	1.8 (1.2–2.6)	66,003 (43,233–96,313)
22	Huangmei	25	1.7 (1.1–2.5)	63,464 (41,180–93,308)
23	Yangxin	24	1.6 (1.0–2.4)	60,926 (39,138–90,293)
24	Daye	20	1.4 (0.8–2.1)	50,772 (31,084–78,123)
25	Gong’an	20	1.4 (0.8–2.1)	50,772 (31,084–78,123)
26	Tongshan	17	1.2 (0.7–1.8)	43,156 (25,192–68,858)
27	Jiayu	16	1.1 (0.6–1.8)	40,617 (23,263–65,737)
28	Zhongxiang	15	1.0 (0.6–1.7)	38,079 (21,353–62,598)
29	Qianjiang	14	0.9 (0.5–1.6)	35,540 (19,465–59,439)
30	Songzi	14	0.9 (0.5–1.6)	35,540 (19,465–59,439)
31	Huarong	13	0.9 (0.5–1.5)	33,001 (17,602–56,257)
32	Zengdu	13	0.9 (0.5–1.5)	33,001 (17,602–56,257)
33	Enshi	12	0.8 (0.4–1.4)	30,463 (15,766–53,051)
34	Liangzihu	12	0.8 (0.4–1.4)	30,463 (15,766–53,051)
35	Zaoyang	11	0.7 (0.4–1.3)	27,924 (13,961–49,817)
36	Dongxihu	10	0.7 (0.3–1.2)	25,386 (12,191–46,553)
37	Wuxue	9	0.6 (0.3–1.2)	22,847 (10,461–43,252)
38	Huangzhou	8	0.5 (0.2–1.1)	20,309 (8,778–39,911)
39	Hannan	8	0.5 (0.2–1.1)	20,309 (8778–39,911)
40	Xian’an	8	0.5 (0.2–1.1)	20,309 (8778–39,911)
41	Xiangzhou	8	0.5 (0.2–1.1)	20,309 (8778–39,911)
42	Zhijiang	8	0.5 (0.2–1.1)	20,309 (8778–39,911)
43	Echeng	7	0.5 (0.2–1.0)	17,770 (7152–36,521)
44	Luotian	7	0.5 (0.2–1.0)	17,770 (7152–36,521)
45	Badong	6	0.4 (0.1–0.9)	15,232 (5595–33,073)
46	Chibi	6	0.4 (0.1–0.9)	15,232 (5595–33,073)
47	Chongyang	6	0.4 (0.1–0.9)	15,232 (5595–33,073)
48	Hongshan	6	0.4 (0.1–0.9)	15,232 (5595–33,073)
49	Shayang	6	0.4 (0.1–0.9)	15,232 (5595–33,073)
50	Shishou	6	0.4 (0.1–0.9)	15,232 (5595–33,073)
51	Sui	6	0.4 (0.1–0.9)	15,232 (5595–33,073)
52	Tuanfeng	6	0.4 (0.1–0.9)	15,232 (5595–33,073)
53	Gucheng	5	0.3 (0.1–0.8)	12,693 (4125–29,554)
54	Xiangcheng	5	0.3 (0.1–0.8)	12,693 (4125–29,554)
55	Jiangling	4	0.3 (0.1–0.7)	10,154 (2769–25,944)
56	Shashi	4	0.3 (0.1–0.7)	10,154 (2769–25,944)
57	Tongcheng	4	0.3 (0.1–0.7)	10,154 (2769–25,944)
58	Yiling	4	0.3 (0.1–0.7)	10,154 (2769–25,944)
59	Yingshan	4	0.3 (0.1–0.7)	10,154 (2769–25,944)
60	Qichun	4	0.3 (0.1–0.7)	10,154 (2769–25,944)
61	Dangyang	3	0.2 (0.04–0.6)	7616 (1571–22,213)
62	Fancheng	3	0.2 (0.04–0.6)	7616 (1571–22,213)
63	Maojian	3	0.2 (0.04–0.6)	7616 (1571–22,213)
64	Xialu	3	0.2 (0.04–0.6)	7616 (1571–22,213)
65	Dongbao	2	0.1 (0.02–0.5)	5077 (615–18,308)
66	Duodao	2	0.1 (0.02–0.5)	5077 (615–18,308)
67	Shannongjia	2	0.1 (0.02–0.5)	5077 (615–18,308)
68	Jianshi	2	0.1 (0.02–0.5)	5077 (615–18,308)
69	Jiang’an	2	0.1 (0.02–0.5)	5077 (615–18,308)
70	Danjiangkou	2	0.1 (0.02–0.5)	5077 (615–18,308)
71	Jingzhou	2	0.1 (0.02–0.5)	5077 (615–18,308)
72	Lichuan	2	0.1 (0.02–0.5)	5077 (615–18,308)
73	Saishan	2	0.1 (0.02–0.5)	5077 (615–18,308)
74	Wuchang	2	0.1 (0.02–0.5)	5077 (615–18,308)
75	Yicheng	2	0.1 (0.02–0.5)	5077 (615–18,308)
76	Yidu	2	0.1 (0.02–0.5)	5077 (615–18,308)
77	Yunxi	2	0.1 (0.02–0.5)	5077 (615–18,308)
78	Changyang	2	0.1 (0.02–0.5)	5077 (615–18308)
79	Zigui	2	0.1 (0.02–0.5)	5077 (615–18308)
80	Baokang	1	0.1 (0.002–0.4)	2538 (64–14,122)
81	Dianjun	1	0.1 (0.002–0.4)	2538 (64–14,122)
82	Dahongshan	1	0.1 (0.002–0.4)	2538 (64–14,122)
83	Qujialing	1	0.1 (0.002–0.4)	2538 (64–14,122)
84	Wujiagang	1	0.1 (0.002–0.4)	2538 (64–14,122)
85	Xiaogan	1	0.1 (0.002–0.4)	2538 (64–14,122)
86	Fang	1	0.1 (0.002–0.4)	2538 (64–14,122)
87	Tieshan	1	0.1 (0.002–0.4)	2538 (64–14,122)
88	Xiling	1	0.1 (0.002–0.4)	2538 (64–14,122)
89	Xianfeng	1	0.1 (0.002–0.4)	2538 (64–14,122)
90	Xingshan	1	0.1 (0.002–0.4)	2538 (64–14,122)
91	Yuan’an	1	0.1 (0.002–0.4)	2538 (64–14,122)
92	Yunyang	1	0.1 (0.002–0.4)	2538 (64–14,122)
93	Zhangwan	1	0.1 (0.002–0.4)	2538 (64–14,122)
94	Zhushan	1	0.1 (0.002–0.4)	2538 (64–14,122)

^1^ ND means that the districts are not divided; CI = confidence interval.

**Table 8:** Joint distribution of Wuhan floating population in Hubei Province.

City	Total	Age Group						Migration Characteristics			Number of Migrant Family Members					Household Registration		Reason for Migration			Education Level		
		<20	21–30	31–40	41–50	51–60	60+	Migration Alone	Nuclear family Migration	Extended Family Migration	1	2	3	4	≥5	Rural	Non-Rural	Work, Business	With Family	Other Reason	Junior High School and Below	High School	College and Above
Total	3,749,479	25,386	1,304,829	1,360,678	751,419	236,088	71,080	487,407	2,985,367	276,705	487,407	548,333	1,627,228	906,272	180,239	3,114,835	632,106	2,891,440	538,178	319,861	1,939,473	1,083,973	726,033
Xiaogan	878,348	7616	279,244	291,936	210,702	71,080	17,770	73,619	738,726	66,003	73,619	152,315	350,324	253,858	48,233	771,728	106,620	662,569	137,083	78,696	540,717	225,933	111,697
Wuhan	733,649	10,154	256,396	286,859	134,545	38,079	7616	93,927	578,796	60,926	93,927	78,696	357,939	170,085	33,002	599,104	134,545	535,640	109,159	88,850	337,631	236,088	159,930
Huanggang	525,486	2539	167,546	208,163	114,236	25,386	7616	68,542	408,711	48,233	68,542	60,926	203,086	149,776	43,156	459,483	66,003	431,558	53,310	40,617	294,475	149,776	81,234
DMP cities	337,631	0	121,852	116,775	68,542	17,770	12,693	48,233	261,473	27,924	48,233	50,772	157,392	66,003	15,231	241,165	96,466	266,551	43,156	27,924	180,239	91,389	66,003
Jingzhou	332,554	2539	106,620	124,390	68,542	25,386	5077	55,849	253,858	22,847	55,849	68,542	129,467	71,080	7616	274,166	58,387	256,396	50,772	25,386	152,315	106,620	73,619
Jingmen	167,546	0	66,003	58,387	30,463	12,693	0	30,463	132,006	5077	30,463	33,002	81,234	20,309	2539	132,006	35,540	139,622	17,770	10,154	78,696	53,310	35,540
Suizhou	149,776	0	66,003	40,617	22,847	15,231	5077	7616	134,545	7616	7616	27,924	76,157	27,924	10,154	132,006	17,770	111,697	38,079	0	81,234	50,772	17,770
Xianning	149,776	0	53,310	60,926	22,847	10,154	2539	27,924	116,775	5077	27,924	20,309	63,464	33,002	5077	129,467	20,309	129,467	7616	12,693	76,157	35,540	38,079
Huangshi	132,006	0	45,694	38,079	35,540	7616	5077	22,847	93,927	15,231	22,847	7616	53,310	38,079	10,154	101,543	30,463	106,620	17,770	7616	73,619	30,463	27,924
Xiangyang	93,927	0	38,079	30,463	17,770	5077	2539	22,847	68,542	2539	22,847	22,847	33,002	12,693	2539	73,619	20,309	68,542	15,231	10,154	33,002	35,540	25,386
Ezhou	86,312	0	38,079	35,540	5077	5077	2539	10,154	73,619	2539	10,154	5077	45,694	22,847	2539	76,157	10,154	55,849	20,309	10,154	43,156	25,386	17,770
Yichang	68,542	0	25,386	33,002	10,154	0	0	5077	58,387	5077	5077	12,693	35,540	15,231	0	43,156	25,386	53,310	10,154	5077	12,693	22,847	33,002
Enshi	58,387	2539	20,309	22,847	7616	2539	2539	12,693	40,617	5077	12,693	5077	25,386	15,231	0	55,849	2539	45,694	10,154	2539	25,386	12,693	20,309
Shiyan	35,540	0	20,309	12,693	2539	0	0	7616	25,386	2539	7616	2539	15,231	10,154	0	25,386	10,154	27,924	7616	0	10,154	7616	17,770

**Table 9:** 2019-nCoV confirmed cases and floating population from Wuhan in Hubei Province.

City	Confirmed Cases in 2020 (Accumulative) ^1^							Floating Population in Wuhan (Unit: 10,000 People) ^2^	Ratio ^3^ (City)
	Jan 25 12:07 p.m.	Jan 26 13:49 p.m.	Jan 27 12:39 p.m.	Jan 28 12:08 p.m.	Jan 29 12:22 p.m.	Jan 30 12:49 p.m.	Jan 31 12:10 p.m.		
DMP cities ^4^	13	16	30	58	77	114	176	33.76	5.21
Xiaogan	26	55	100	173	274	399	541	87.83	6.16
Jingzhou	10	33	47	71	101	151	221	33.26	6.65
Huanggang	64	122	154	213	324	496	573	52.55	10.90
Xianning	0	43	64	91	112	130	166	14.98	11.08
Huangshi	0	31	36	53	86	113	168	13.20	12.73
Enshi	11	17	25	38	51	66	75	5.84	12.85
Jingmen	21	38	90	114	142	191	227	16.75	13.55
Suizhou	5	36	52	70	116	143	228	14.98	15.22
Ezhou	1	1	20	57	84	123	189	8.63	21.90
Yichang	1	20	31	51	63	117	167	6.85	24.36
Xiangyang	0	8	36	70	131	163	286	9.39	30.45
Shiyan	5	20	40	65	88	119	150	3.55	42.21
Pr ^5^	0.65	0.61	0.67	0.78	0.80	0.81	0.84	-	-

^1^ The number of confirmed cases was from Chinese dynamic epidemic monitoring website (A confirmed case refers to a suspected case who have tested positive via a nucleic acid test); ^2^ The number of floating populations from Wuhan is calculated value (Please see Table 6); ^3^ Ratio = Confirmed cases (on 2020/1/31)/Floating population from Wuhan (Unit: 10,000 people); ^4^ DMP (Directly Managed by the Province) cities includes Xiantao, Qianjiang and Tianmen; ^5^ The Pearson’s correlation coefficient is calculated from the number of floating populations in Wuhan and the number of confirmed cases per day.

**Table 10:** 2019-nCoV confirmed cases and floating population from Wuhan in China.

Province	Confirmed Cases in 2020 (Accumulative)							Floating Population in Wuhan (Unit: 10,000 People)	Ratio (Province)
	Jan 25 12:07 p.m.	Jan 26 13:49 p.m.	Jan 27 12:39 p.m.	Jan 28 12:08 p.m.	Jan 29 12:22 p.m.	Jan 30 12:49 p.m.	Jan 31 12:10 p.m.		
Qinghai	1	1	4	6	6	6	8	0.80	10.00
Xizang	0	0	0	0	0	1	1	0.10	10.00
Henan	32	83	128	168	206	278	352	33.75	10.43
Gansu	4	7	14	19	24	26	29	2.10	13.81
Guizhou	4	5	7	9	9	12	15	0.95	15.79
Anhui	39	60	70	106	152	200	237	13.65	17.36
Jiangxi	18	36	48	72	109	162	240	12.15	19.75
Fujian	10	18	35	59	82	101	120	5.00	24.00
Chongqing	57	75	110	132	147	165	206	8.25	24.97
Sichuan	15	44	69	90	108	142	177	6.95	25.47
Hunan	43	69	100	143	221	277	332	12.60	26.35
Shanxi	6	9	13	20	27	35	39	1.20	32.50
Hebei	8	13	18	33	48	65	82	2.40	34.17
Jilin	4	4	6	8	9	14	14	0.40	35.00
Jiangsu	18	31	47	70	99	129	168	4.60	36.52
Heilongjiang	9	15	21	30	37	43	59	1.55	38.06
Guangxi	23	33	46	51	58	78	87	1.75	49.71
Shandong	21	46	63	87	121	145	178	3.10	57.42
Shaanxi	5	22	35	35	56	63	87	1.50	58.00
Liaoning	12	19	23	30	36	41	45	0.70	64.29
Zhejiang	62	104	128	173	296	428	537	6.95	77.27
Xinjiang	3	4	5	10	13	14	17	0.20	85.00
Guangdong	78	98	146	188	241	311	393	3.05	128.85
Yunnan	5	11	19	26	44	70	76	0.45	168.89
Inner Mongolia	1	7	11	13	16	18	20	0.10	200.00
Tianjin	8	13	17	24	25	28	32	0.15	213.33
Hainan	8	22	31	40	43	46	50	0.20	250.00
Beijing	36	54	68	80	91	114	132	0.40	330.00
Ningxia	2	4	7	11	12	17	21	0.05	420.00
Shanghai	33	40	53	66	80	101	128	0.15	853.33
Pr (All province)	0.40	0.59	0.63	0.66	0.62	0.62	0.63	-	-
Pr (First type)	0.90	0.91	0.94	0.96	0.96	0.96	0.96	-	-
Pr (Second type)	0.56	0.72	0.76	0.78	0.70	.69	0.70	-	-
Pr (Second type excluded Henan and Zhejiang)	0.78	0.83	0.81	0.86	0.89	0.91	0.93	-	-
